# Association of MASP2 levels and MASP2 gene polymorphisms with systemic lupus erythematosus

**DOI:** 10.1111/jcmm.15656

**Published:** 2020-07-17

**Authors:** Wang‐Dong Xu, Xiao‐Yan Liu, Lin‐Chong Su, An‐Fang Huang

**Affiliations:** ^1^ Department of Evidence‐Based Medicine Southwest Medical University Luzhou China; ^2^ Department of Rheumatology and Immunology Hubei Minzu University Enshi China; ^3^ Department of Rheumatology and Immunology Affiliated Hospital of Southwest Medical University Luzhou China

**Keywords:** autoimmunity, lupus, MASP2

## Abstract

Systemic lupus erythematosus (SLE) is a chronic inflammatory autoimmune disorder. MASP2 is a mediator that plays an important role in complement system. As dysregulation of the complement system has been demonstrated to correlate with SLE pathogenesis, the role of MASP2 in lupus has not been widely discussed. In the present study, serum levels of MASP2 were evaluated in 61 lupus patients and 98 healthy controls by training cohort, and then a validation cohort including 100 lupus, 100 rheumatoid arthritis, 100 osteoarthritis, 100 gout, 44 Sjogren's syndrome, 41 ankylosing spondylitis patients confirmed the findings. Receiver operating characteristic (ROC) curve analysis determined the discriminatory capacity for serum MASP2. PCR methods tested the association of *MASP2* gene polymorphisms (rs7548659, rs17409276, rs2273346, rs1782455 and rs6695096) and SLE risk. Impact of polymorphism on MASP2 serum levels was evaluated as well. Results showed that serum levels of MASP2 were significantly higher in lupus patients and correlated with some clinical, laboratory characteristics in the training cohort, and were much higher as compared to that in different rheumatic diseases patients in the validation cohort. Serum MASP2 showed a good diagnostic ability for lupus. Genotype frequencies and allele frequency of polymorphisms rs7548659, rs2273346 were strongly related to SLE risk, and genotypes of rs17409276, rs1782455, rs76695096 were significantly correlated with lupus genetic susceptibility. Interestingly, patients carrying GA genotype of rs17409276, TT, TC genotype of rs6695096 showed higher levels of serum MASP2. The findings suggested that MASP2 may be a potential disease marker for lupus, and correlate with SLE pathogenesis.

## 1 INTRODUCTION

Systemic lupus erythematosus (SLE) is a systemic autoimmune disease with chronic inflammation and tissues, organs damage. Genetics and dysregulated immunity have been found related to pathogenesis of SLE.^1^

Complement system plays an important role in innate immunity and has been recognized to bridge innate and adaptive immune system.^2^ It is known that complement is able to clear up immune complexes, apoptotic cells and efficiently regulate pro‐inflammatory components production in response to pathogens.^3^ Lectin pathway activates the complement, which will be further activated via mannose‐binding lectin (MBL)‐associated serine protease (MASPs). MASPs are the enzymatic constituents of the lectin pathway of the complement system. The MASPs family has three subgroups, including MASP1, MASP2 and MASP3. MASP1 was discovered as a bactericidal factor with structural similarities to C1s, while the biological function of MASP3 has not been clearly elucidated to date.^4^ The *MASP2* gene is located on chromosome 1p36.3‐36.2, encoding MASP2 and MAp19. There are 12 exons for *MASP2* gene. It is notable that exon 2 encodes the signal peptide. Along with exon 3, both of exon 2 and 3 encode CUB1 domain. Exon 4 encodes the epidermal growth factor‐like domain, and exon 12 encodes serine protease domain, 3’ UTR region.^5^ MASP2 binds to MBL, ficolins, forming a homodimer, which autoactivates and initiates the lectin pathway.^6^ In addition, MASP2 cleaves prothrombin, further leading to the covalent bind of cross‐linked fibrin on bacterial surfaces.^7^ As numerous studies indicated that the lectin pathways are dysregulated in SLE patients, evidence about MASP2 protein expression in SLE patients and association of *MASP2* gene polymorphisms and SLE risk is limited. Therefore, in the present study, we discussed serum levels of MASP2 in lupus patients with large samples and revealed the SLE genetic susceptibility with *MASP2* single nucleotide polymorphism (SNP).

## 2 METHODS

### 2.1 Patients

A total of 250 SLE patients (222 female and 28 male, age 37.72 ± 13.44 years) in Chinese Han population were recruited from the department of Rheumatology and Immunology in affiliated hospital of Southwest Medical University, Minda hospital of Hubei Minzu University. SLE was diagnosed with 1982 American College of Rheumatology (ACR) criteria.^8^ Another 385 age and sex‐matched healthy volunteers were collected as controls (345 female and 40 male, age 38.87 ± 9.42 years). The present study has two parts. Within the first part, MASP2 serum levels were evaluated. Interestingly, we firstly evaluated MASP2 serum levels in a training cohort by 61 SLE patients and 98 healthy controls so as to discuss whether there is a difference between patients and controls. Then, the validation cohort with another 100 SLE patients was used to confirm the findings in training cohort, along with 100 rheumatoid arthritis (1987 ACR revised criteria for RA),^9^ 100 osteoarthritis (OA) (Osteoarthritis Criteria Subcommittee of the American Rheumatism Association),^10^ 100 gout (1977 American Rheumatism Association),^11^ 44 Sjogren's syndrome (American‐European classification criteria),^12^ 41 ankylosing spondylitis patients (Modified New York criteria).^13^ In the second part, we genotyped 5 SNPs (rs7548659, rs17409276, rs2273346, rs1782455 and rs6695096) of *MASP2* gene in order to discuss the genetic susceptibility of SLE with *MASP2* variations. This study was admitted by Ethics Research Committee of Southwest Medical University, and consent was collected from individual participant because of Declaration of Helsinki. Clinical and laboratory data were obtained from all the subjects (Table 1).

**TABLE 4:** The demographic and clinical characteristics of SLE patients and controls

Characteristics	SLE	Healthy controls	*P* value
Female (%)/male (%)	88.80/11.20	89.61/10.39	.705
Age (mean ± SD, y)	37.72 ± 13.44	38.87 ± 9.42	.256
LN [n (%)]	115 (46.00)	—	
Lupus headache [n (%)]	16 (6.40)	—	
Vasculitis [n (%)]	18 (7.20)	—	
Arthritis [n (%)]	108 (43.20)	—	
Myositis [n (%)]	12 (4.80)	—	
Rash [n (%)]	102 (40.80)	—	
Alopecia [n (%)]	64 (25.60)	—	
Oral ulcer [n (%)]	28 (11.20)	—	
Pleuritis [n (%)]	23 (9.20)	—	
Pericarditis [n (%)]	22 (8.80)	—	
Fever [n (%)]	45 (18.00)	—	
Hypocomplementemia [n (%)]	131 (52.40)	—	
ds‐DNA (+) [n (%)]	60 (24.00)	—	
Thrombocytopenia [n (%)]	34 (13.60)	—	
Reduced leukocyte [n (%)]	26 (10.40)	—	
Cylindruria [n (%)]	12 (4.80)	—	
Hematuria [n (%)]	85 (34.00)	—	
Pyuria [n (%)]	22 (8.80)	—	

Abbreviations: LN, lupus nephritis; SLE, systemic lupus erythematosus.

### 2.2 Serum, DNA preparation and genotyping

Peripheral blood was collected from cubital vein from participants, further centrifuged for serum obtainment, stored at −80℃ until usage. Peripheral blood mononuclear cell (PBMC) was obtained by density gradient centrifugation as well. Genomic DNA was extracted using TIANamp Blood DNA kits (Tiangen). Concentration and purity qualified DNA were further tested for genotyping of *MASP2* gene polymorphisms (rs7548659, rs17409276, rs2273346, rs1782455 and rs6695096) by KASP (Gene Company).^14^ KASP primers were summarized in Table S1.

### 2.3 Measurement of MASP2 by Enzyme‐linked immune sorbent assay (ELISA)

Concentrations of serum MASP2 were determined by ELISA for both patients and controls. The kits for human MASP2 were purchased from Cusabio^15^ and evaluated the protein according to the manufactory.

### 2.4 Statistics

All the data were analysed by SPSS 16.0 and STAT 11.0. If the data were normally distributed, mean ± standard deviation (SD) was used to describe the data, and Student's *t* test, ANOVA was appropriately used to test the differences between groups. Otherwise, median and inter‐quartile range were selected and non‐parametric test determined the differences between groups. Spearman's rank correlation tested relation between two variables. Hardy‐Weinberg equilibrium (HWE) examined the genotypes distribution of individual polymorphism in healthy controls. Receiver operating characteristic (ROC) curve discussed the potential of serum MASP2 as the biomarker for lupus. Statistical power was assessed by power and sample size calculation version 3.1.6 software (http://biostat.mc.vanderbilt.edu/PowerSampleSize). *P* value less than .05 was recognized significant.

## 3 RESULTS

### 3.1 MASP2 serum levels in training cohort

**FIGURE 1:**
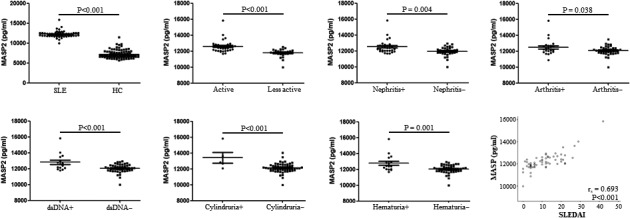
Serum levels of MASP2 in lupus patients from training cohort. Expression of serum MASP2 was tested by Enzyme‐linked immune sorbent assay (ELISA) in 61 systemic lupus erythematosus (SLE) patients and 98 healthy controls. Every symbol represents an independent lupus patient and healthy control. Student's *t* test was used to discuss the difference between two groups. Spearman's nonparametric test evaluated correlation between serum levels of MASP2 and SLE disease activity index

In training cohort, there were 61 lupus patients and 98 age, sex‐matched healthy controls, and serum levels of MASP2 were significantly higher in lupus patients as compared to that in controls (12 230.52 ± 779.65 vs 7174.45 ± 999.45 pg/mL, *P* < .001, Figure 1). According to subgroup analysis with SLE disease activity index (SLEDAI) score, patients with active disease (N = 34) had elevated serum levels of MASP2 as compared to that in less active (N = 27) patients (12 595.28 ± 770.33 vs 11 771.20 ± 506.43 pg/mL, *P* < .001). SLE patients with nephritis (N = 30) revealed higher expression of MASP2 than that in patients without nephritis (N = 31) (12 518.77 ± 847.03 vs 11 951.58 ± 598.88 pg/mL, *P* = .004). Patients with arthritis (N = 23) had elevated serum levels of MASP2 when compared with that in patients without arthritis (N = 38) (12 494.59 ± 984.27 vs 12 070.70 ± 583.17 pg/mL, *P* = .038). Similarly, lupus patients with positive anti‐dsDNA (N = 15) revealed increased expression of MASP2 in serum than that in patients with negative anti‐dsDNA antibody (N = 46) (12 825.41 ± 1074.12 vs 12 036.54 ± 543pg/mL, *P* < .001). Lupus patients with cylindruria (N = 5) also had elevated serum levels of MASP2 than that in patients without the parameter (N = 56) (13 414.07 ± 1472.66 vs 12 124.85 ± 605.53 pg/mL, *P* < .001). Moreover, patients with hematuria (N = 16) showed increased serum levels of MASP2 compared with the patients without hematuria (N = 45) (12 789.44 ± 1054.64 vs 12 031.80 ± 543.80pg/mL, *P* = .001). The other clinical, laboratory characteristics were not related to MASP2 expression in lupus patients (data not shown). Correlation analysis found that serum levels of MASP2 were strongly correlated with SLEDIA score (r_s_ = 0.693, *P* < .001, Figure 1).

### 3.2 Increased serum levels of MASP2 in validation cohort

**FIGURE 2:**
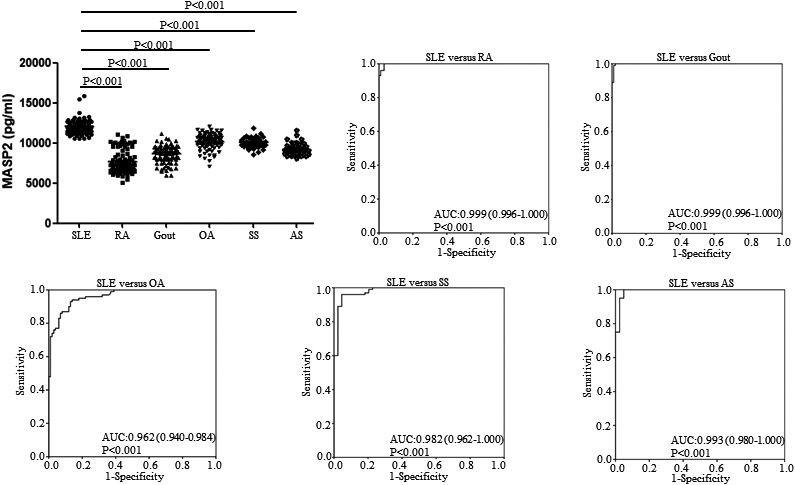
Validation of serum MASP2 in SLE patients. Serum levels of MASP2 were examined by Enzyme‐linked immune sorbent assay (ELISA) in 100 SLE patients and 100 rheumatoid arthritis (RA), 100 osteoarthritis (OA), 100 gout, 44 Sjogren's syndrome (SS), 41 ankylosing spondylitis (AS) patients. Serum levels of MASP2 were used to distinguish SLE from other rheumatic diseases (RA, OA, gout, SS, AS) by receiver‐operating characteristic curve analysis

As the serum levels of MASP2 were higher in SLE patients and related to several clinical, laboratory characteristics as shown in the training cohort, it is possible that dysregulated serum MASP2 may be a disease marker for lupus. To further confirm the potential of serum MASP2 as a biomarker of lupus, we conducted a validation cohort that included 100 SLE patients, 100 RA patients, 100 OA patients, 100 gout patients, 44 SS patients and 41 AS patients. Results showed that lupus patients showed significantly up‐regulated serum levels of MASP2 when compared to that in different rheumatic patients (All *P* < .001, Figure 2). ROC analysis indicated that area under curve (AUC) was 0.999 when serum levels of MASP2 in lupus patients compared to that in RA patients. Similarly, serum MASP2 in lupus patients compared with that in gout, OA, SS and AS patients revealed AUC of 0.999, 0.962, 0.982, 0.993, respectively (Figure 2).

### 3.3 *MASP2* gene polymorphisms with lupus patients’ genetic susceptibility

In the present study, a total of 250 lupus patients and 385 age, sex‐matched healthy volunteers were selected to discuss the *MASP2* gene polymorphisms (rs7548659, rs17409276, rs2273346, rs1782455 and rs6695096) and disease susceptibility. Genotypes frequencies of all the polymorphisms in healthy volunteers were according with HWE (Table S2). In addition, the powers were 0.923 for rs7548659, 0.831 for rs17409276, 0.865 for rs2273346, 0.753 for rs1782455 and 0.881 for rs6695096 to detect a 1.9‐fold increased risk assuming an *α* value of 0.05. With respect to rs7548659, frequency of genotype TT was significantly different between lupus patients and controls (odds ratio (OR) = 2.059, 95% confidence interval (CI): 1.094‐3.877, *P* = .025 when TT vs GG) (Table 2). Allele T comparing with allele G revealed OR = 1.424, 95% CI: 1.108‐1.830, *P* = .006. For rs17409276, frequency of GG vs AA between patients and controls showed OR = 1.943, 95% CI: 1.046‐3.610, *P* = .036; OR = 2.267, 95% CI: 1.162‐4.423, *P* = .016 (GA vs AA); OR = 2.028, 95% CI: 1.101‐3.736, *P* = .023 (GG + GA vs AA). For rs2273346, OR = 2.027, 95% CI: 1.029‐3.991, *P* = .041 (TT vs CC); OR = 1.368, 95% CI: 1.042‐1.795, *P* = .024 (allele T vs allele C). For rs1782455, OR = 2.498, 95% CI: 1.014‐6.150, *P* = .046 (TT vs CC); OR = 2.831, 95% CI: 1.112‐7.206, *P* = .029 (TC vs CC); OR = 2.585, 95% CI: 1.056‐6.330, *P* = .038 (TT + TC vs CC). For rs76695096, OR = 2.055, 95% CI: 1.102‐3.831, *P* = .023 (TT vs CC); OR = 1.978, 95% CI: 1.030‐3.796, *P* = .040 (TC vs CC); OR = 2.028, 95% CI: 1.101‐3.736, *P* = .023 (TT + TC vs CC) (Table 2).

**TABLE 1:** Allele and genotype frequencies of five single nucleotide polymorphisms in MASP2 gene in SLE patients and healthy controls

Polymorphism	SLE (n)	Ctl (n)	OR (95% CI)	*P* value
rs7548659				
TT	117	220	2.059 (1.094‐3.877)	.025
TG	110	144	1.434 (0.755‐2.723)	.271
TT + TG	227	364	1.756 (0.950‐3.246)	.072
GG	23	21	—	
T	344	584	1.424 (1.108‐1.830)	.006
G	156	186	—	
rs17409276				
GG	166	258	1.943 (1.046‐3.610)	.036
GA	59	107	2.267 (1.162‐4.423)	.016
GG + GA	225	365	2.028 (1.101‐3.736)	.023
AA	25	20	—	
G	391	623	1.181 (0.895‐1.560)	.240
A	109	147	—	
rs2273346				
TT	148	255	2.027 (1.029‐3.991)	.041
TC	82	113	1.621 (0.800‐3.285)	.180
TT + TC	230	368	1.882 (0.966‐3.668)	.063
CC	20	17	—	
T	378	623	1.368 (1.042‐1.795)	.024
C	122	147	—	
rs1782455				
TT	175	269	2.498 (1.014‐6.150)	.046
TC	62	108	2.831 (1.112‐7.206)	.029
TT + TC	237	377	2.585 (1.056‐6.330)	.038
CC	13	8	—	
T	412	646	1.113 (0.824‐1.502)	.485
C	88	124	—	
rs76695096				
TT	146	240	2.055 (1.102‐3.831)	.023
TC	79	125	1.978 (1.030‐3.796)	.040
TT + TC	225	365	2.028 (1.101‐3.736)	.023
CC	25	20	—	
T	371	605	1.275 (0.979‐1.660)	.071
C	129	165	—	

Abbreviations: 95% CI, 95% confidence interval; OR, odd ratio.

### 3.4 Correlation between *MASP2* gene polymorphisms with lupus clinical, laboratory parameters

As discussed above, the five polymorphisms in *MASP2* gene were significantly related to SLE genetic susceptibility. To further evaluate the association of *MASP2* gene polymorphisms and lupus clinical, laboratory parameters, subgroup analysis was tested for each polymorphism. Results showed that lupus patients with thrombocytopenia having increased frequencies of TT genotype, allele T as compared to the patients without the parameter for rs7548659 (*P* = .010, *P* = .004, respectively). Patients with pyuria also revealed increased frequencies of TT genotype, allele T as compared to the patients without the parameter (*P* = .039, *P* = .022) (Table 3). For rs17409276, patients with the clinical, laboratory characteristics including pleuritis, pericarditis, hypocomplementemia, positive anti‐dsDNA, pyuria showed much higher frequencies of genotype GG, allele G than that in lupus patients did not have the characteristics (Table 3). Similarly, frequencies of genotype TT, allele T for rs2273346 were higher in patients with rash, and frequencies of genotype TT, allele T for rs1782455 were increased in lupus patients having fever, thrombocytopenia, pyuria when compared with patients without the characteristics, respectively (Tables 3 and 4). Furthermore, patients had oral ulcer, hypocomplementemia showed elevated frequencies of genotype TT + TC, allele T as compared to that in patients without oral ulcer, hypocomplementemia for rs6695096 (Table 4).

**TABLE 2:** Analysis of MASP2 gene polymorphisms (rs7548659, rs17409276 and rs2273346) in SLE by clinical, laboratory features.

Characteristics	rs7548659							rs17409276							rs2273346						
	Genotype (n)			*P*	Allele (n)		*P*	Genotype (n)			*P*	Allele (n)		*P*	Genotype (n)			*P*	Allele (n)		*P*
	TT	TG	GG		T	G		GG	GA	AA		G	A		TT	TC	CC		T	C	
Lupus nephritis																					
Positive	56	50	9	.736^a^	162	68	.466^b^	75	27	13	.815^a^	177	53	.239^b^	67	40	8	.749^a^	174	56	.980^b^
Negative	61	60	14		182	88		91	32	12		214	56		81	42	14		204	66	
Lupus headache																					
Positive	7	7	2	.786^a^	21	11	.689^b^	9	5	2	.672^a^	23	9	.370^b^	10	6	0	.410^a^	26	6	.442^b^
Negative	110	103	21		323	145		157	54	23		368	100		138	76	20		352	116	
Vasculitis																					
Positive	8	10	0	.353^a^	26	10	.062^b^	11	6	0	.246^a^	28	6	.572^b^	11	7	0	.410^a^	29	7	.472^b^
Negative	109	100	23		318	246		155	53	24		363	101		137	75	20		349	115	
Arthritis																					
Positive	55	47	6	.178^a^	157	59	.102^b^	73	24	11	.905^a^	170	46	.812^b^	71	27	10	.071^a^	169	47	.231^b^
Negative	62	63	17		187	97		93	35	14		221	63		77	55	10		209	75	
Myositis																					
Positive	6	5	1	.974^a^	17	7	.826^b^	7	4	1	.717^a^	18	6	.697^b^	5	7	0	.124^a^	17	7	.577^b^
Negative	111	105	22		327	149		159	55	24		373	103		143	75	20		361	115	
Rash																					
Positive	46	50	6	.207^a^	142	62	.746^b^	65	26	11	.759^a^	156	48	.437^b^	70	27	5	.034^a^	167	37	.007^b^
Negative	71	60	17		202	94		101	33	14		235	61		78	55	15		211	85	
Alopecia																					
Positive	25	35	4	.126^a^	85	43	.498^b^	40	17	7	.744^a^	97	31	.442^b^	35	23	6	.685^a^	93	35	.369^b^
Negative	92	75	19		259	113		126	42	18		294	78		113	59	14		285	87	
Oral ulcer																					
Positive	10	15	3	.458^a^	35	21	.195^b^	16	9	3	.497^a^	41	15	.338^b^	19	7	2	.603^a^	45	11	.379^b^
Negative	107	95	20		329	135		150	50	22		350	94		129	75	18		333	111	
Pleuritis																					
Positive	11	10	2	.993^a^	32	14	.906^b^	11	5	7	.003^a^	27	19	.001^b^	15	5	3	.386^a^	35	11	.936^b^
Negative	106	100	21		312	142		155	54	18		364	90		133	77	17		343	111	
Pericarditis																					
Positive	10	11	1	.679^a^	31	13	.804^b^	12	4	6	.018^a^	28	16	.014^b^	14	6	2	.843^a^	34	10	.787^b^
Negative	107	99	22		313	143		154	55	19		363	93		134	76	18		344	12	
Fever																					
Positive	24	19	2	.389^a^	67	23	.202^b^	29	9	7	.363^a^	67	23	.341^b^	26	16	2	.607^a^	68	20	.670^b^
Negative	93	91	21		277	133		137	50	18		324	86		121	66	18		308	102	
Hypocomplementemia																					
Positive	58	60	13	.693^a^	176	86	.411^b^	79	29	23	<.001^a^	187	75	<.001^b^	73	50	8	.122^a^	196	66	.666^b^
Negative	59	50	10		168	70		87	30	2		204	34		75	32	12		182	56	
ds‐DNA (+)																					
Positive	26	28	6	.825^a^	80	40	.563^b^	35	14	11	.044^a^	84	36	.013^b^	39	18	3	.466^a^	96	24	.198^b^
Negative	91	82	17		264	116		131	45	14		307	73		109	64	17		282	98	
Thrombocytopenia																					
Positive	24	9	1	.010^a^	57	11	.004^b^	25	5	4	.418^a^	55	13	.564^b^	19	14	1	.337^a^	52	16	.779^b^
Negative	93	101	22		287	145		141	54	21		336	96		129	68	19		316	106	
Reduced leukocyte																					
Positive	12	11	3	.908^a^	35	17	.806^b^	15	9	2	.372^a^	39	13	.555^b^	16	7	3	.680^a^	39	13	.915^b^
Negative	105	99	20		309	139		151	50	23		352	96		132	75	17		339	109	
Cylindruria																					
Positive	9	2	1	.117^a^	20	4	.115^b^	7	3	2	.278^a^	17	7	.370^b^	8	4	0	.569^a^	20	4	.366^b^
Negative	108	108	22		324	152		159	56	23		374	102		140	78	20		358	118	
Hematuria																					
Positive	41	36	8	.931^a^	118	52	.832^b^	55	18	12	.278^a^	128	42	.259^b^	52	29	4	.387^a^	133	37	.325^b^
Negative	76	74	15		226	104		111	41	13		263	67		96	53	16		245	85	
Pyuria																					
Positive	16	5	1	.039^a^	37	7	.022^b^	21	1	0	.010^a^	43	1	.001^b^	14	7	1	.800^a^	35	9	.523^b^
Negative	101	105	22		307	149		145	58	25		348	108		134	75	19		343	113	

^a^ Patients positive vs patients negative using 3 × 2 contingency table.

^b^ Patients positive vs patients negative using 2 × 2 contingency table.

**TABLE 3:** Analysis of MASP2 gene polymorphisms (rs1782455 and rs6695096) in SLE by clinical, laboratory features

Characteristics	rs1782455							rs6695096						
	Genotype (n)			*P*	Allele (n)		*P*	Genotype (n)			*P*	Allele (n)		*P*
	TT	CT	CC		T	C		TT	TC	CC		T	C	
Lupus nephritis														
Positive	82	27	6	.904^a^	191	39	.727^b^	65	39	11	.767^a^	169	61	.734^b^
Negative	93	35	7		221	49		81	40	14		202	68	
Lupus headache														
Positive	11	5	0	.554^a^	27	5	.762^b^	9	5	2	.941^a^	23	9	.756^b^
Negative	164	57	13		385	83		137	74	23		348	120	
Vasculitis														
Positive	11	7	0	.249^a^	29	7	.763^b^	12	4	2	.673^a^	28	8	.611^b^
Negative	164	55	13		383	81		134	75	23		343	121	
Arthritis														
Positive	75	25	8	.368^a^	175	41	.479^b^	60	38	10	.565^a^	158	58	.639^b^
Negative	100	37	5		237	47		86	41	15		213	71	
Myositis														
Positive	7	4	1	.653^a^	18	6	.329^b^	9	3	0	.363^a^	21	3	.127^b^
Negative	168	58	12		394	82		133	76	25		350	126	
Rash														
Positive	68	28	6	.633^a^	164	40	.328^b^	63	30	9	.659^a^	156	48	.335^b^
Negative	107	34	7		248	48		83	49	16		215	81	
Alopecia														
Positive	43	18	3	.769^a^	104	24	.710^b^	32	26	6	.193^a^	90	38	.244^b^
Negative	132	44	10		306	64		114	53	19		281	91	
Oral ulcer														
Positive	17	10	1	.356^a^	44	12	.425^b^	10	14	4	.034^a^	34	22	.014^b^
Negative	158	52	12		368	76		136	65	21		337	107	
Pleuritis														
Positive	16	5	2	.708^a^	37	9	.713^b^	15	8	0	.245^a^	38	8	.171^b^
Negative	159	57	11		375	79		131	71	25		333	121	
Pericarditis														
Positive	15	4	3	.159^a^	34	10	.303^b^	16	5	1	.338^a^	37	7	.116^b^
Negative	160	57	10		377	75		130	74	24		334	122	
Fever														
Positive	36	9	0	.126^a^	81	9	.037^b^	28	12	5	.731^a^	68	22	.746^b^
Negative	139	53	13		331	79		118	67	20		303	107	
Hypocomplementemia														
Positive	90	30	11	.053^a^	210	52	.166^b^	83	39	9	.126^a^	205	57	.030^b^
Negative	85	32	2		202	36		63	40	16		166	72	
ds‐DNA (+)														
Positive	41	14	5	.452^a^	96	24	.428^b^	40	17	3	.213^a^	97	23	.060^b^
Negative	134	48	8		316	64		106	61	22		272	105	
Thrombocytopenia														
Positive	29	5	0	.053^a^	63	5	.017^b^	17	11	6	.249^a^	45	23	.104^b^
Negative	146	57	13		349	83		129	68	19		326	106	
Reduced leukocyte														
Positive	16	9	1	.466^a^	41	11	.477^b^	19	5	2	.268^a^	43	9	.135^b^
Negative	159	53	12		371	77		127	74	23		326	120	
Cylindruria														
Positive	9	3	0	.704^a^	21	3	.475^b^	6	5	1	.744^a^	17	7	.699^b^
Negative	166	59	13		381	85		140	74	24		354	122	
Hematuria														
Positive	61	17	7	.171^a^	139	31	.789^b^	47	28	10	.687^a^	122	48	.372^b^
Negative	114	45	6		273	57		99	51	15		249	81	
Pyuria														
Positive	21	1	0	.024^a^	43	1	.005^b^	10	9	3	.433^a^	29	15	.188^b^
Negative	154	61	13		369	87		136	70	22		342	114	

^a^ Patients positive vs patients negative using 3 × 2 contingency table.

^b^ Patients positive vs patients negative using 2 × 2 contingency table.

### 3.5 Impact of *MASP2* gene polymorphisms on serum levels

**FIGURE 3:**
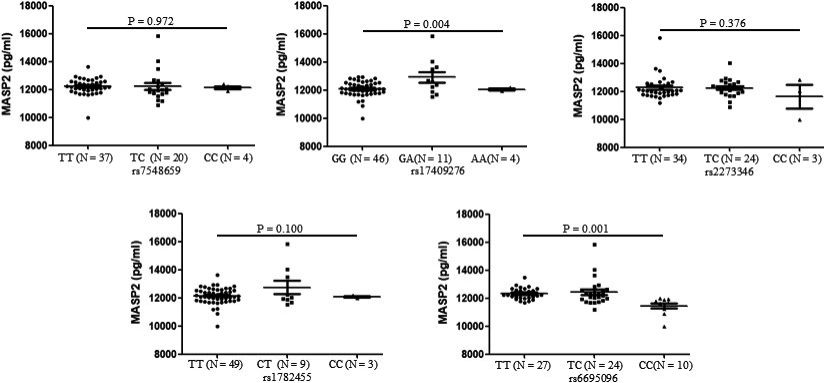
Correlation between *MASP2* gene polymorphisms and serum expression in lupus patients. *MASP2* gene polymorphisms (rs7548659, rs17409276, rs2273346, rs1782455 and rs6695096) were genotyped by PCR in SLE patients. Comparison of MASP2 values among three groups was performed by Kruskal‐Wallis test

To discuss the possible role of *MASP2* gene polymorphisms on serum levels of MASP2 in lupus patients, we analysed the correlation between polymorphisms and serum levels of MASP2. Results showed that patients had comparable expression of MASP2 for rs7548659 (*P* = .972), for rs2273346 (*P* = .376), for rs1782455 (*P* = .100) when discussing the expression of MASP2 among three groups of genotypes (Figure 3). However, lupus patients carrying genotypes GG, GA, AA for rs17409276, TT, CT, CC for rs6695096 had significantly different serum levels of MASP2, by which carrying GA genotype for rs17409276, carrying TT, TC genotype revealed higher expression of MASP2 (Figure 3).

## 4 DISCUSSION

This study is the first time to report serum levels of MASP2 in lupus patients with large sample size. We firstly compared serum levels of MASP2 in lupus patients and healthy controls by the training cohort and found that there was elevated expression of MASP2 in SLE. Increased expression of MASP2 was related to clinical, laboratory characteristics including nephritis, arthritis, anti‐dsDNA antibody, cylindruria, hematuria and SLEDAI score. Then, a validation cohort demonstrated the serum levels of MASP2 in SLE, showing with significantly higher expression as compared to that in RA, gout, OA, SS, AS patients. Interestingly, ROC curve analysis revealed that serum levels of MASP2 had good diagnosis ability. Collectively, these data indicated that MASP2 may correlate with SLE pathogenesis and may be a potential biomarker for SLE.

Compared with previous findings, there is limited evidence about MASP2 in autoimmune diseases especially association with lupus. Only a study with 58 female lupus patients showed that plasma levels of MASP2 were not significantly different from that in healthy controls.^16^ The differences between this study and our findings may correlate with several reasons. First, different sample size may relate to different results where we had larger sample size, showing more credible conclusion. Second, in our case‐control study, there were about 90% female and about 10% male patients; however, the study published by Troldborg et al recruited all female patients. Therefore, different ratio of female and male patients in a study may correlate with the differences. Third, lupus patients collected in the study designed by Troldborg et al had different treatment such as hydroxychloroquine, prednisolone, mycophenolate mofetil and azathioprine. On the contrary, patients in our study were all treatment naïve. It is reasonable that plasma levels of MASP2 were comparable between lupus patients and controls in the study by Troldborg et al, and increased serum levels of MASP2 in our study may indicate the dysregulated immunity in lupus patients, and the up‐regulated inflammatory response in lupus. Fourth, different reagents to detect the protein expression of MASP2 in lupus patient may have different results. When discussed the relationship of serum MASP2 levels and clinical, laboratory features, patients with nephritis, arthritis, anti‐dsDNA antibody, cylindruria, hematuria showed higher expression of MASP2 as compared to that in lupus patients without the characteristics. Interestingly, studies discussing association of MASP2 and arthritis showed that plasma levels of MASP2 were higher than that in synovial fluid in RA patients, and ratio of synovial fluid/plasma concentration was increased in RA patients compared to OA patients.^17^ *MASP2* gene deficient (MASP2‐/‐) mice treated with bovine collagen type II protected arthritis induction, evidenced by reduced clinical disease activity, decreased histopathological scores, C3 deposition and infiltration of synovial macrophages, neutrophils, whereas the wild‐type mice showed severe features of arthritis.^18^ It is possible that increased MASP2 in lupus patients may correlate to the development of arthritis, promoting disease severity like the role of MASP2 in arthritis pathogenesis. However, the clear role of MASP2 contributing to lupus clinical, laboratory features such as arthritis needs discussion in the future.

Polymorphism in a gene can affect transcription to mRNA and then affect translation of protein expression. Therefore, discussing *MASP2* gene polymorphism is of importance to reveal the lupus genetic susceptibility. In this study, we recruited 250 lupus patients and matched with 385 sex, age comparable healthy volunteers to discuss the *MASP2* rs7548659, rs17409276, rs2273346, rs1782455 and rs6695096 polymorphisms. We found that TT genotype, T allele of rs7548659, rs2273346 was related to SLE risk, while GG, GG + GA genotypes of rs17409276, TT, TC, TT + TC genotypes of rs1782455, rs76695096 were strongly correlated with lupus susceptibility. To further reveal the association of *MASP2* gene polymorphisms with lupus clinical, laboratory features, we did subgroup analysis. Findings showed that different polymorphisms related to different features. Recent studies have discussed association of *MASP2* gene polymorphisms and human diseases, such as RA, tuberculosis. In a study with RA patients from Brazil, two *MASP2* haplotypes CCTGGCCCC (rs7548559(C) + rs61735600(C) + rs72550870(T) + rs56392418(G) + rs17409276(G) + rs12711521(C) + rs2273346(C) + rs12085877(C) + rs1782455(C)), CCTGGACCC (rs7548559(C) + rs61735600(C) + rs72550870(T) + rs56392418(G) + rs17409276(G) + rs12711521(A) + rs2273346(C) + rs12085877(C) + rs1782455(C)) up‐regulated susceptibility to RA, and allele T of rs72550870, allele C of rs12085877 correlated with articular symptoms in RA patients.^19^ Genotype TC at rs2273346 and rs6695096 of *MASP2* genes were more prevalent in the tuberculosis patients in Canada than the healthy controls.^20^ These findings suggested that the *MASP2* gene polymorphisms may correlate with inflammatory diseases including SLE. However, further studies with larger sample sizes and different ethnicities are needed to confirm the observations in our study.

It is notable that patients with positive anti‐dsDNA antibody had higher frequency of GG as compared to the patients with negative anti‐dsDNA antibody for rs17409276, and patients with positive anti‐dsDNA antibody had higher serum levels of MASP2, suggesting that rs17409276 polymorphism in *MASP2* gene may affect MASP2 protein expression and play a role in SLE pathogenesis. Therefore, we analysed the association of different gene polymorphisms and MASP2 protein expression in lupus patients from the training cohort. We found that genotypes of rs7548569, rs2273346, rs1782455 were not significantly related to MASP2 protein expression, whereas patients carrying GA genotype of rs17409276 had higher serum levels of MASP2 when compared to the patients carrying AA genotype, patients carrying TT genotype or TC genotype of rs6695096 had higher serum levels of MASP2 as compared to the patients carrying CC genotype. These data indicated that genotype GA of rs17409276, genotypes TT, TC may affect *MASP2* variations, and therefore promoted production of MASP2 in lupus patients. However, what mechanisms did polymorphisms regulate the expression of MASP2 is still needed elucidation.^21^

Some limitations in this study should be realized. First, gene polymorphisms were not examined in other group of patients. Second, functional role of MASP2 involves in SLE pathogenesis should be discussed in the future. Third, a larger sample size to evaluate association of *MASP2* gene polymorphisms with SLE risk will be better to reveal the genetic susceptibility of SLE.

In conclusion, this study found that serum levels of MASP2 may correlate with SLE pathogenesis, and *MASP2* gene polymorphisms related to SLE genetic susceptibility in a Chinese Han population.

## CONFLICT OF INTEREST

None.

## AUTHOR CONTRIBUTION

**Wang‐Dong Xu:** Writing‐review & editing (equal). **Xiao‐Yan Liu:** Investigation (supporting). **Lin‐chong Su:** Methodology (supporting). **An‐Fang Huang:** Writing‐review & editing (equal).

## Supporting information

Table S1

Table S2

## Data Availability

Datasets are available from the corresponding author on reasonable request.
