# Supplementary material for: Association of MASP2 levels and MASP2 gene polymorphisms with systemic lupus erythematosus
Source: J Cell Mol Med. 2020 Jul 17;24(18):10432–43. doi: 10.1111/jcmm.15656 (PMC7521335; doi:10.1111/jcmm.15656)
Supplement: Supplementary file 1 — Table S1 [file JCMM-24-10432-s001.doc]

Supplementary table 1 Hardy-Weinberg’s expectation test in healthy controls of the polymorphism

| Polymorphism |  | Healthy controls | |
| --- | --- | --- | --- |
| rs7548659 |  | χ^2^=0.335 | P=0.846 |
| rs17409276 |  | χ^2^=1.969 | P=0.374 |
| rs2273346 |  | χ^2^=0.067 | P=0.967 |
| rs1782455 |  | χ^2^=0.797 | P=0.671 |
| rs76695096 |  | χ^2^=0.470 | P=0.791 |
