# Supplementary material for: Association of MASP2 levels and MASP2 gene polymorphisms with systemic lupus erythematosus
Source: J Cell Mol Med. 2020 Jul 17;24(18):10432–43. doi: 10.1111/jcmm.15656 (PMC7521335; doi:10.1111/jcmm.15656)
Supplement: Supplementary file 2 — Table S2 [file JCMM-24-10432-s002.docx]

Supplementary Table 2 The primer information for MASP2 gene polymorphisms

| Polymorphism | Primer_AlleleFAM | Primer_AlleleHEX | Primer_Common |
| --- | --- | --- | --- |
| rs7548659 | GGCAGGACAGGCTGCTCTG | CAGGCAGGACAGGCTGCTCTT | CCAGGATTTGTTTCCAGGCTGTTGTT |
| rs17409276 | AAGGCAGCACACCTCTTCCTCT | GGCAGCACACCTCTTCCTCC | GAACTGAGGGGGAGTCGTCCAT |
| rs2273346 | GATCTACCCAGTGGCCGAGC | ATGATCTACCCAGTGGCCGAGT | GTGGTCACTCCAGGACCTGTGAT |
| rs1782455 | GTCTATGAGCAAAAACATGATGCATCT | CTATGAGCAAAAACATGATGCATCC | GGCTTGTGTATAATGAGGTGATAGTCTTT |
| rs6695096 | CCAAAGCTGTGCTCTCACAGC | CCCAAAGCTGTGCTCTCACAGT | AGCACCTCGTAAAACACTAGAATAGGAA |
